# Colostrum-Derived Melatonin Plus PEG Microspheres Modulate the Oxidative Metabolism of Human Colostrum Phagocytes

**DOI:** 10.3390/metabo15010057

**Published:** 2025-01-16

**Authors:** Caroline G. Silva, Viviane F. Luz, Victor L. Nunes, Ana B. M. Verzoto, Aron C. de M. Cotrim, Wagner B. dos Santos, Eduardo L. França, Adenilda C. Honorio-França

**Affiliations:** 1 Programa de Pós-Graduação em Ciência de Materiais, Campus Universitário do Araguaia, Universidade Federal de Mato Grosso, Barra do Garças 78605-091, Brazil; gomescaroline@outlook.com (C.G.S.); vivianefrancelina@gmail.com (V.F.L.); aroncarlosbg@gmail.com (A.C.d.M.C.); wbsantos32@yahoo.com.br (W.B.d.S.); 2 Instituto de Ciências Biológicas e da Saúde, Campus Universitário do Araguaia, Universidade Federal de Mato Grosso, Barra do Garças 78605-091, Brazil; victor.nunes@sou.ufmt.br (V.L.N.); beatrizmverzoto@gmail.com (A.B.M.V.)

**Keywords:** hormone, colostrum, modified release, superoxide, superoxide dismutase

## Abstract

Background/Objectives: Exogenous melatonin adsorbed onto PEG microspheres can modulate the functional activity of phagocytes in colostrum, but no data are available on the activity of melatonin found in colostrum. Therefore, the objective of this study was to extract melatonin from human colostrum, develop and characterize PEG microspheres with the extracted melatonin adsorbed onto them, and evaluate the effects of this system on the oxidative metabolism of colostrum phagocytes. Methods: Thirty colostrum samples were collected; ten were used for melatonin extraction, while twenty were used to obtain phagocytes. Melatonin was extracted from the colostrum supernatant through affinity chromatography and quantified by ELISA. The polyethylene glycol microspheres produced were analyzed using fluorescence microscopy and flow cytometry. Oxidative metabolism was assessed by measuring the release of the superoxide anion and superoxide enzymes. A control was conducted using commercial melatonin. Results: The fluorescence microscopy and flow cytometry analyses demonstrated that PEG microspheres can adsorb melatonin. There was an increase in superoxide release in phagocytes incubated with colostrum-derived or synthetic melatonin. When exposed to bacteria, colostrum phagocytes treated with colostrum melatonin adsorbed to PEG microspheres exhibited increased superoxide, accompanied by a decrease in the release of superoxide dismutase (SOD) and a lower SOD-to-superoxide ratio. In contrast, synthetic melatonin reduced the release of superoxide and increased the release of the enzyme and the SOD-to-superoxide ratio. Conclusions: These data highlight the importance of melatonin on cellular metabolism and suggest that colostrum-derived melatonin may be a more effective option for controlling oxidative metabolism, particularly during infectious processes.

## 1. Introduction

Modified release systems enhance the body’s ability to use drugs more efficiently than traditional methods. They optimize drug distribution, minimize fluctuations in concentration, and improve bioavailability. These systems influence the release speed, pharmacokinetic profile, site of action, and duration of effects while potentially reducing side effects [1,2,3].

Polymers are a key type of modified release system. These large molecules are made of repeating units called monomers, consisting of carbon, hydrogen, and other non-metallic elements. An example is polyethylene glycol (PEG) microspheres, known for their biocompatibility and water solubility. PEG is derived from ethylene oxide, and its properties vary with chain lengths and molecular weight. The microspheres can adsorb drugs within their PEG matrix and are adjustable sizes for different applications [4,5].

PEG microspheres have been linked to melatonin to enhance its bioavailability and prevent degradation, thus improving the immune response [6,7]. Melatonin is a neurohormone found in blood and colostrum. It plays a crucial role in cellular processes, acts on cellular signaling pathways, and possesses antioxidant properties that influence various bodily functions [8,9]. Additionally, melatonin may directly impact bacterial infections, affecting microorganisms and the host’s immune response, depending on treatment, the immunopineal axis’s physiological response, anti-inflammatory action at the beginning of the infection, and the adaptive immune response [10].

Melatonin, produced by the pineal gland, is highly hydrophobic, leading to an oral bioavailability of less than 20% due to hepatic metabolism and variable absorption rates [5,7]. PEG microspheres, a biocompatible and amphiphilic material, offer a promising modified melatonin release system. This system enables gradual release over 24 h and improves stability, solubility, and bioavailability [7,8].

PEG microspheres combined with the hormone melatonin can modulate the activity of colostrum phagocytes in the presence of Enteropathogenic *Escherichia coli* (EPEC), one of the main causes of enteric infections in children [11,12]. Melatonin enhances the functional activity of these phagocytes, highlighting the importance of colostrum’s bioactive components for immune protection [13]. While colostrum and human milk are known to combat infections, research on the effects of colostrum-derived melatonin on cellular oxidative metabolism is lacking. Considering that colostrum and human milk effectively combat intestinal and respiratory infections, it is possible that the melatonin present in these secretions interacts with the mononuclear (MN) phagocytes and enhances their functional activity against bacterial infections. This study aimed to extract melatonin from human colostrum, develop and characterize PEG microspheres with the extracted melatonin adsorbed onto them, and evaluate the effects of this system on the oxidative metabolism of colostrum phagocytes.

## 2. Materials and Methods

### 2.1. Subjects and Sample Size

A total of 30 healthy mothers assisted by the Public Health System were evaluated for obtaining colostrum samples. The study included postpartum women between the ages of 18 and 35 who gave birth to a healthy baby without any malformations, had a gestational age at delivery between 37 and 41^6/7^ weeks, who tested negative for syphilis, hepatitis B, hepatitis C, and HIV, did not use continuous medication, and had no chronic conditions such as obesity, hypertension, or diabetes.

The sample size proposed in this study was based on statistical calculations of the sample size assuming a loss to follow-up of around 10% and correcting for the effects of errors α (5%) and β (20%) attributed to the study.

**Figure 1:**
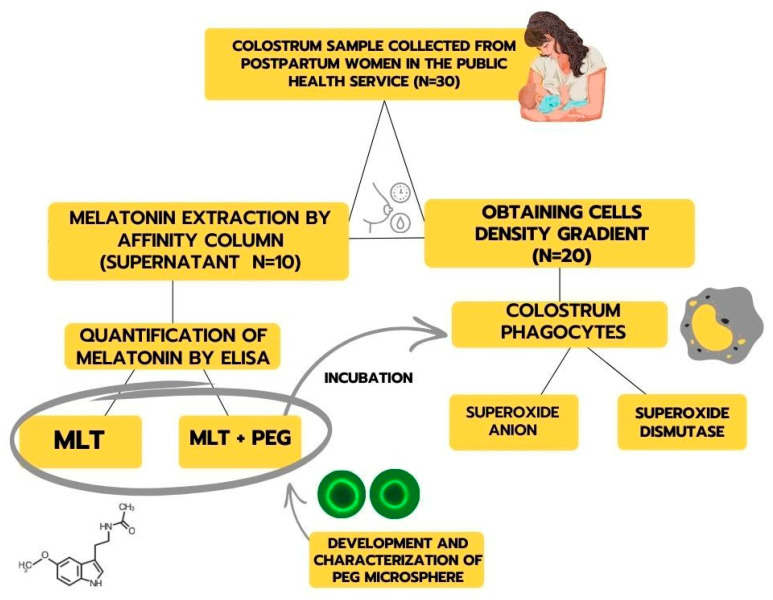
Representative scheme for obtaining samples and experimental design.

Mothers were asked about their willingness to donate a colostrum sample (approximately 8 mL). The sample was collected manually, using an appropriate technique, in the morning and between two feedings, corresponding to the first 48 to 72 h after delivery. The scheme for obtaining samples and experimental design is described in Figure 1.

### 2.2. Obtaining the Human Colostrum Supernatant Pool

Ten colostrum samples were used to obtain the colostrum supernatant. The colostrum supernatant was obtained by centrifugation for 10 min at 160× *g* at 4 °C. After obtaining the colostrum supernatant, the samples were mixed and centrifuged for 10 min at 300× *g* to obtain the human colostrum pool. This resulting supernatant pool was used for melatonin extraction. All samples were pre-tested for possible contamination before melatonin extraction.

### 2.3. Extraction and Quantification of the Colostrum Melatonin Hormone

The Melatonin ELISA Kit (IBL) was used to extract and quantify melatonin in colostrum via affinity chromatography. The columns were set in glass tubes, centrifuged with 1 mL of methanol for 1 min at 200 g, then washed twice with double-distilled water. After preparing the columns, 0.5 mL of standards, controls, and samples were applied and centrifuged. The columns were washed with 1.0 mL of 10% methanol, and the eluate was extracted using 1.0 mL of methanol. This eluate was concentrated by evaporating methanol in a Speed-Vac and reconstituted with 0.15 mL of double-distilled water.

For ELISA analysis, 50 µL of each sample, standard, and control were added to an ELISA plate with 50 µL of melatonin–biotin and 50 µL of antibody, then incubated at 4 °C. After discarding the supernatant and washing the plate, 150 µL of the conjugated enzyme was added and incubated at room temperature for 120 min. Following three washes, 200 µL of the substrate p-nitrophenyl phosphate (PNPP) was added and agitated for 40 min. The reaction was stopped with 50 µL of PNPP stop solution, and absorbance was measured at 405 nm. Results were calculated based on the standard curve and expressed in pg/mL.

### 2.4. Preparation of Polyethylene Glycol (PEG) Microspheres

PEG 6000 (Sigma-St Louis, St Louis, MO, USA), was used to produce PEG microspheres, following the protocol established by Scott et al. [14] and modified by Scherer et al. [15]. Initially, 2 g of PEG 6000 was resuspended in 100 milliliters of phosphate-buffered saline (PBS) and incubated at 37 °C for 30 min in a water bath. An equal volume of a 2% sodium sulfate solution in PBS was added, and the samples were incubated at 37 °C for 45 min. The samples were then diluted in a 3:1 ratio with PBS and centrifuged at 5000 RPM for 2 min. After a further 1:10 dilution in PBS, the mixture was incubated at 37 °C for 25 min. Microsphere formation occurred by heating to 95 °C for 5 min.

For adsorption, the suspensions of microspheres in PBS were incubated with melatonin extracted from colostrum and synthetic melatonin hormone from Sigma, both at 100 pg/mL, at 37 °C for 30 min.

### 2.5. Characterization of PEG Microspheres by Fluorescence Microscopy and Flow Cytometry

PEG microspheres, adsorbed or not with colostrum melatonin and synthetic melatonin, were labeled with a DyLight 488 solution diluted in dimethylformamide (10 µg/mL–Pierce) overnight at room temperature, using a molar ratio of 100:1. The experiments were conducted three times, and results were analyzed through fluorescence SE microscopy (Nikon Eclipse, Tokyo, Japan).

The size of the PEG microspheres was measured with a FACSCalibur Flow Cytometer (Becton Dickinson (BD), San José, CA, USA) and compared to commercial polymethylacrylate microspheres (6 µm—CaliBRITE 3 Beads, BD Cat. No. 340486, USA). For incubation, PEG microspheres were treated with 5 µL of phycoerythrin (PE–Sigma) for 30 min at 37 °C, with or without added melatonin. Following this, they were washed in PBS with Bovine Serum Albumin (BSA—5 mg/mL; 500 rpm, 10 min, 4 °C). Flow cytometry confirmed the size and fluorescence intensity of the PEG microspheres, calculated as the geometric mean of Forward Scatter (FSC). All experiments were performed in triplicate, and the figures present the results.

### 2.6. Release of Melatonin from Colostrum and Synthetic Adsorbed in PEG Microsphere

After adsorbing melatonin from colostrum and synthetic sources (100 pg/mL) onto PEG microspheres, the preparation was resuspended in RPMI culture medium to assess the release of melatonin. The melatonin adsorbed onto the PEG microspheres, both from colostrum (PEG-MLT-C) and synthetic sources (PEG-MLT-S), was incubated for 0, 2, 6, 12, 18, and 24 h at 37 °C in a 5% CO_2_ environment. After each incubation period, the suspensions of PEG-MLT were centrifuged for 10 min at 160× *g*, and the supernatant was collected for melatonin quantification. The melatonin release was determined using an ELISA kit (Immune-Biological Laboratories, Hamburg, Germany). Reaction rates were measured using an absorbance plate-reading spectrophotometer with a 405 nm filter. The results were calculated based on the standard curve and reported in pg/mL.

### 2.7. Obtaining Mononuclear Cells from Human Colostrum

Twenty samples of colostrum were centrifuged for 10 min at 1500 rpm to separate the cells. The supernatant was discarded, and the cell pellet was resuspended in 5 mL medium 199. Cells were then separated using a density gradient (Ficoll-Paque—density 1.077 -Cytiva, Marlborough, MA, USA) for 40 min at 1500 rpm at 4 °C.

The ring of mononuclear cells that formed between the layers was carefully transferred to a new centrifuge tube using a Pasteur pipette. Next, 3 mL of glucose-containing PBS was added to the tube, and the sample was homogenized to perform the first wash. After discarding the supernatant, a second wash with PBS was conducted. Finally, 1 mL of glucose-containing PBS was added and homogenized with the cell pellet. The cell concentration was adjusted to 2 × 10^6^ cells/mL using a Neubauer chamber.

### 2.8. Enteropathogenic Escherichia coli (EPEC) Strands and Cultures

Enteropathogenic *Escherichia coli* (EPEC) serotype 0111: H^−^AL^−^, eae^+^, eaf^+^, bfp^+^, stored at −70 °C, were used. The stock culture was maintained on semi-solid agar in the absence of light. From this stock culture, 18 h before the test, subcultures were made in tubes containing 8 mL of TSB (Tryptic Soy Broth—Difco), and these were incubated in an oven at 37 °C for 18 h. After growth, the bacteria were washed twice in phosphate-buffered saline (PBS), and the concentration was adjusted to 1 × 10^7^ bacteria per mL, measured with a spectrophotometer (540 nm, OD = 0.1).

### 2.9. Incubation of MN Phagocytes with PEG Microspheres Adsorbed with Melatonin Extracted from Colostrum and Synthetic Melatonin

The MN cells from the colostrum were maintained in culture for 2 h in the presence or absence of melatonin extracted from colostrum or synthetic melatonin (Sigma, St Louis, MO, USA) adsorbed or not on PEG microspheres, in the presence or absence of EPEC, in an incubator at 37 °C with 5% CO_2_. After this period, the cells were used in the superoxide anion assays, and the culture supernatant was reserved for quantification of the superoxide enzyme (SOD). For each assay performed, as a control of the experiments, phagocytes (2 × 10^6^ cells/mL) were incubated for a similar time, depending on the type of assay, in medium 199 or PBS, in the absence of melatonin.

### 2.10. Superoxide Anion Release

The release of superoxide anion from colostrum phagocytes was analyzed using ferricytochrome C to assess cellular oxidative metabolism, following the method of Pick and Mizel [16]. In the presence of superoxide anion, ferricytochrome C is oxidized to ferrocytochrome C, which can be detected colorimetrically with a spectrophotometer.

Cells were separated, adjusted to a concentration of 2 × 10^6^ cells/mL, and centrifuged at 160 G for 10 min. With or without PEG microspheres adsorbed with either colostrum-derived or synthetic melatonin, equal volumes of bacteria and cells were incubated for 15 min at 37 °C to allow phagocytosis. The mixture was centrifuged again, and excess extracellular bacteria were removed before resuspending in 0.5 mL of glucose-containing PBS with ferricytochrome C at 2 mg/mL. A control containing only cells was prepared to measure spontaneous superoxide anion release. The suspensions were incubated at 37 °C for 1 h before measuring optical density at 630 nm. The superoxide anion concentration was calculated using the formula: O_2_^–^ concentration (nmol) = Optical Density (OD) × 100/6.3.

### 2.11. CuZn-Superoxide Dismutase in the Supernatant Culture of Colostrum Phagocytes

The activity of CuZn-superoxide dismutase (CuZn-SOD—E.C.1.15.1.1) was assessed using the nitroblue tetrazolium (NBT) photoreduction method. After the incubation of MN phagocytes with or without polyethylene glycol (PEG) microspheres adsorbed with melatonin, extracted from colostrum or synthetic, in the presence or not of bacteria, the samples were centrifuged at 160 G for 10 min, and the supernatant was retained. Following this, 0.5 mL of a hydroalcoholic solution, 0.5 mL of a chloroform–ethanol solution (1:1), and 0.5 mL of a mixture containing NBT and EDTA were added to each tube, along with 2.0 mL of carbonate buffer, adjusting the pH to 10.2 with hydroxylamine. The tubes stood at room temperature for 15 min. Absorbance was measured at 560 nm, corresponding to the reduced form of NBT. CuZn-SOD activity was calculated based on the reduction of NBT, with the activity determined from the difference in absorbance between the sample and the control.

### 2.12. Statistical Analysis

The data were organized in Excel@ and are presented as the mean ± standard deviation (SD). Statistically significant differences in superoxide anion, superoxide dismutase, and superoxide dismutase/superoxide anion ratio were assessed with the BioEstat^®^ version 5.0 software [Mamirauá Institute, Belém, Brazil]. A D’Agostino normality test and analysis of variance (ANOVA) were used for the statistical analysis of the data, followed by Tukey’s means comparison test. Statistical significance was considered for a *p*-value less than 0.05 (*p* < 0.05).

## 3. Results

### 3.1. Characterization of PEG Microspheres

After extracting melatonin from colostrum, 24 eluates with varying hormone concentrations were produced. The total yield from the extraction was 1375 ng per 10 mL. The hormone concentration was adjusted to 100 pg/mL for the adsorption of melatonin into the PEG microsphere.

**Figure 2:**
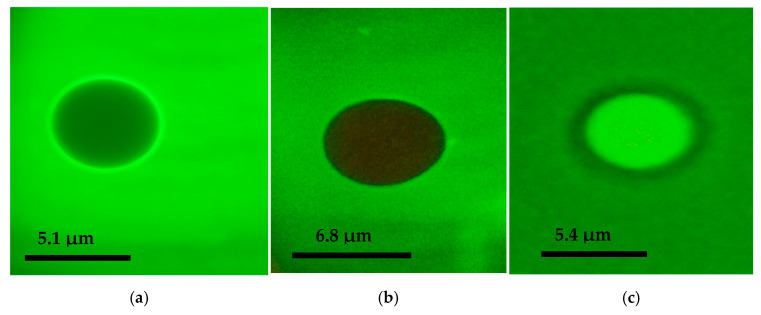
Fluorescence microscopy images of PEG microspheres stained with DyLight 488 (1000×): (**a**) represents the PEG microsphere without melatonin adsorption, (**b**) represents the microsphere incubated with melatonin extracted from colostrum, and (**c**) represents the microsphere incubated with synthetic melatonin.

The polyethylene glycol microspheres produced were analyzed by fluorescence microscopy and flow cytometry. Fluorescence microscopy demonstrated that PEG microspheres can effectively adsorb melatonin extracted from colostrum in addition to synthetic melatonin (Figure 2b,c). The PEG microspheres exhibited spherical structures with consistent and stable sizes. However, the PEG microspheres that adsorbed melatonin from colostrum (Figure 2b) were larger compared to those that adsorbed synthetic melatonin (Figure 2c) and those that did not adsorb any melatonin (Figure 2a).

**Figure 3:**
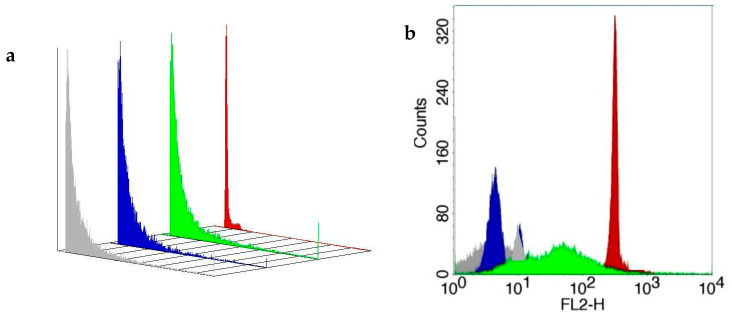
Three-dimensional image of size (panel (**a**)) and fluorescence intensity (FL-2; panel (**b**)) and of PEG microspheres adsorbed or not with melatonin extracted from colostrum stained with phycoerythrin (PE) as described in Materials and Methods. The standard PE-labeled polymethylmethacrylate microsphere (BD Microsphere, Becton Dickinson, San Jose, CA, USA) was used as a standard (FACScalibur, Becton Dickinson, San Jose, USA). Flow cytometry (FACScalibur, Becton Dickinson, USA) evaluated microsphere size and immunofluorescence analysis. Gray color—PEG microsphere; blue color—BD microsphere; green color—microsphere plus synthetic melatonin; red—microsphere plus colostrum melatonin.

Flow cytometry analysis revealed that PEG microspheres with melatonin extracted from colostrum had a size of approximately 6.8 µm. The PEG microsphere adsorbed with synthetic melatonin had a size of approximately 5.4 µm compared to BD Polymethylmethacrylate microspheres (6 µm) (Table 1). Polyethylene glycol microspheres adsorbed or not with melatonin extracted from colostrum with PE-labeled microspheres are shown in Figure 3a,b.

**Figure 4:**
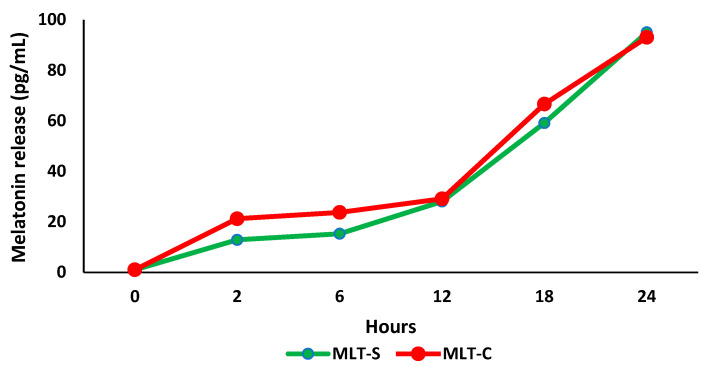
Melatonin release (pg/mL) from colostrum and synthetic melatonin in polyethylene glycol (PEG) microspheres during 24 h of incubation in RPMI 1640 medium (n = 3). Melatonin from colostrum (MLT-C); synthetic melatonin (MLT-S).

Figure 4 demonstrates the release of melatonin—from colostrum and synthetic sources—adsorbed onto PEG microspheres over 24 h. The release rate increased over time, and after 24 h, more than 90% of both types of melatonin had been released, with concentrations of 93.2 pg/mL for melatonin from colostrum (MLT-C) and 95.1 pg/mL for synthetic melatonin (MLT-S). The mean of release was 39.1 pg/mL for melatonin from colostrum (MLT-C) and 35.2 pg/mL for synthetic melatonin (MLT-S).

### 3.2. Effect of Melatonin Adsorbed to PEG Microspheres on Superoxide Anion Release

**Figure 5:**
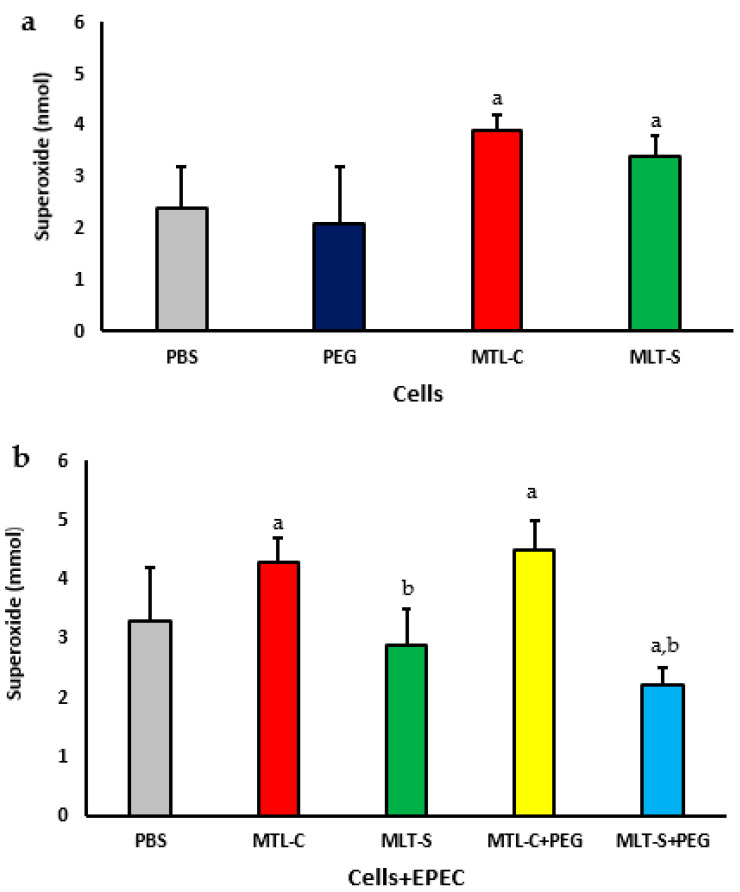
Superoxide release from human colostrum MN phagocytes. Results represent the mean and standard deviation of 20 colostrum samples. Cells were treated with PEG microspheres, colostrum melatonin (MLT-C), or synthetic melatonin (MLT-S) (**a**). They were also incubated with enteropathogenic *Escherichia coli* (EPEC) and then treated with PEG microspheres containing either MLT-C or MLT-S (**b**). The letter “a” indicates a significant difference (*p* < 0.05) in superoxide anion release between the PBS-treated group and those treated with MLT-C and MLT-S. Letter “b” shows differences between MLT-C-PEG and MLT-S-PEG treatments.

Figure 5 illustrates the concentrations of superoxide released by colostrum phagocytes, with or without PEG microspheres, that are adsorbed with human colostrum melatonin or synthetic. It was observed that phagocytes incubated with both types of melatonin (colostrum or synthetic) increased superoxide release. Phagocytes incubated only with PEG exhibited superoxide release levels similar to the spontaneous release. Still, these levels were lower than those observed with PEG microspheres with both adsorbed melatonin types (Figure 5a).

When colostrum melatonin was adsorbed onto PEG microspheres, an increase in the release of superoxide anions by colostrum phagocytes was observed, regardless of whether the phagocytes were incubated with bacteria and in contrast to the phagocytes that were incubated with PEG microspheres adsorbed with synthetic melatonin (see Figure 5b) and in the presence of EPEC; colostrum phagocytes that were incubated with PEG microspheres containing synthetic melatonin exhibited a decrease in the release of superoxide anion compared to phagocytes incubated only with the bacteria (see Figure 5b).

### 3.3. Effect of Melatonin Adsorbed to PEG Microspheres on CuZn-Superoxide Dismutase (SOD) Enzyme Activity

**Figure 6:**
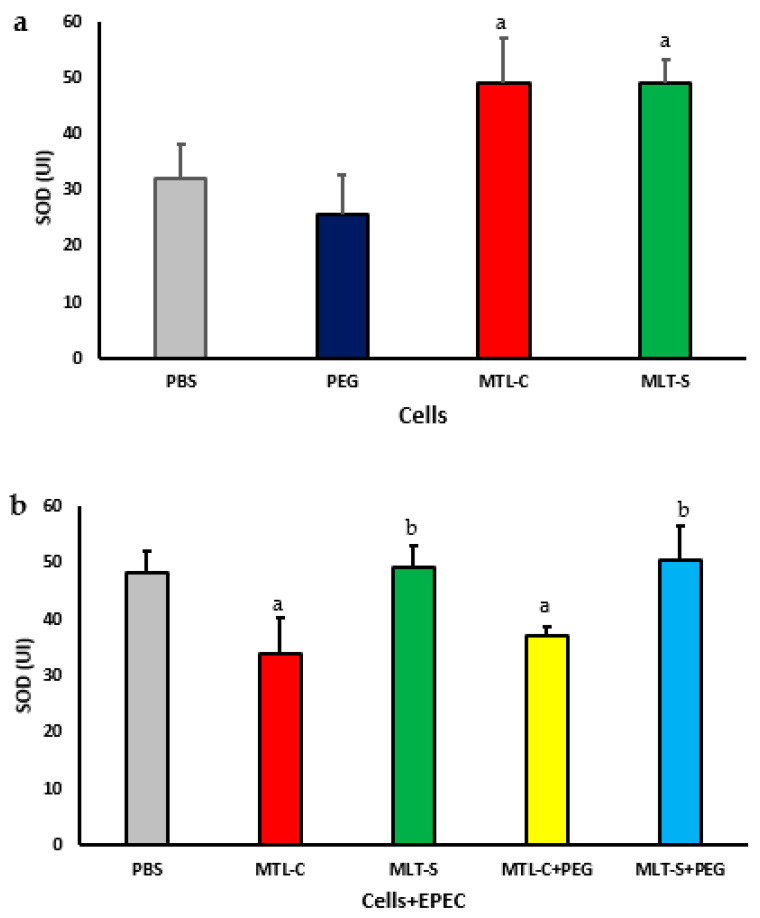
The concentration of CuZn-superoxide dismutase (SOD) in the culture supernatant of mononuclear (MN) phagocytes from human colostrum was analyzed using 20 samples. MN cells were treated with colostrum-derived and synthetic melatonin (**a**). Additionally, MN cells exposed to enteropathogenic *Escherichia coli* (EPEC) were treated with PEG microspheres containing colostrum-derived or synthetic melatonin (**b**). Results showed significance at *p* < 0.05. The letter “a” highlights differences in enzyme levels between the PBS group and those treated with colostrum-derived melatonin (MLT-C), synthetic melatonin (MLT-S), or PEG microspheres (MLT-C-PEG and MLT-S-PEG). Letter “b” indicates the differences between the PEG microspheres with colostrum melatonin (MLT-C-PEG) and those with synthetic melatonin (MLT-S-PEG).

The concentration of the superoxide dismutase (SOD) enzyme in the culture supernatants of colostrum phagocytes in the presence of both melatonin extracted from colostrum and synthetic melatonin is illustrated in Figure 6a. It is evident that, regardless of the type of melatonin used, there was an increase in the enzyme levels in both culture supernatants. The supernatant from cells treated with PEG microspheres demonstrated SOD concentrations similar to those of untreated cells (Figure 6a).

Figure 6b illustrates the concentration of superoxide dismutase (SOD) enzyme in the culture supernatant of colostrum phagocytes in the presence of EPEC. The phagocytes were treated with microspheres containing melatonin extracted from colostrum or synthetic melatonin. The results showed that, regardless of the presence of microspheres, the melatonin extracted from colostrum resulted in lower concentrations of SOD. In contrast, the synthetic melatonin, whether adsorbed onto the microsphere or not, led to higher concentrations of SOD (Figure 6b). The lowest concentrations of SOD were found in the culture supernatant treated with colostrum-derived melatonin.

**Figure 7:**
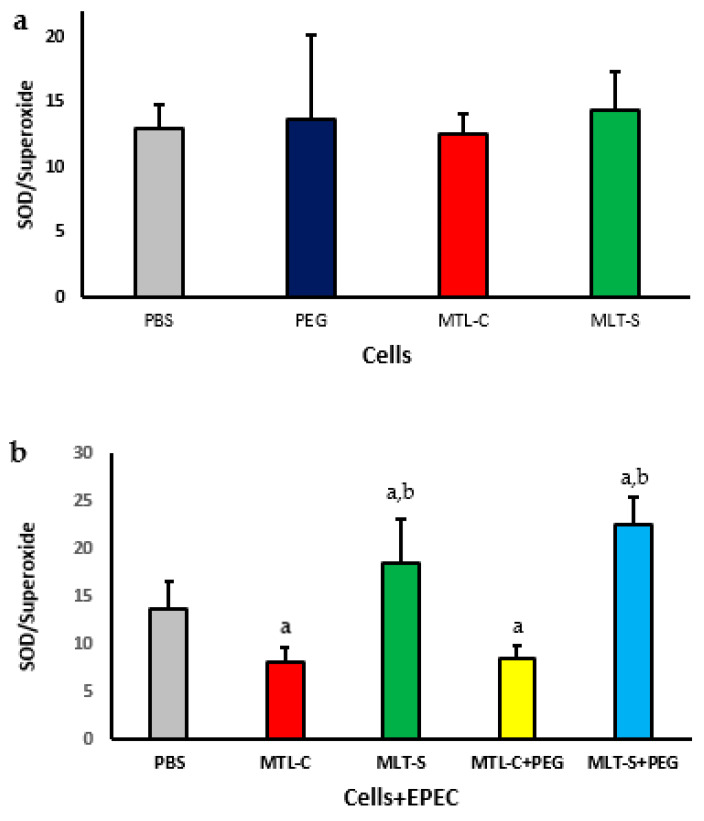
SOD/superoxide ratio in human colostrum MN phagocytes with colostrum melatonin and synthetic melatonin (**a**). Results represent the mean and standard deviation of 20 colostrum samples. It also presents the ratio for MN phagocytes exposed to EPEC (enteropathogenic *Escherichia coli*) and treated with PEG microspheres containing either type of melatonin (**b**), with *p* < 0.05. The letter “a” highlights differences in enzyme levels between the PBS group and those treated with colostrum-derived melatonin (MLT-C), synthetic melatonin (MLT-S), or PEG microspheres (MLT-C-PEG and MLT-S-PEG). Letter “b” indicates the differences between the PEG microspheres with colostrum melatonin (MLT-C-PEG) and those with synthetic melatonin (MLT-S-PEG).

The SOD/O_2_^−^ ratio in the supernatant of cultures containing human colostrum cells treated with melatonin extracted from colostrum or in synthetic form, with or without being adsorbed onto PEG microspheres in the presence or absence of EPEC, is presented in Figure 7. It is noted that colostrum phagocytes showed no significant difference in the SOD/O_2_^−^ ratio when exposed to either colostrum-derived melatonin or synthetic melatonin (Figure 7a).

The SOD/O_2_^−^ ratio of colostrum cells decreased in the presence of EPEC, regardless of whether they were treated with melatonin extracted from colostrum adsorbed onto PEG microspheres. However, treatment with synthetic melatonin, regardless of the presence of PEG microspheres, resulted in an increased SOD/O_2_^−^ ratio, with values higher than those observed in the group treated with PBS. The highest SOD/superoxide indices were found in the group of cells treated with PEG microspheres that contained adsorbed synthetic melatonin (Figure 7b).

## 4. Discussion

In this study, PEG microspheres were produced and loaded with colostrum melatonin. This system modulated the cellular oxidative metabolism in the presence of bacteria, as indicated by changes in the release of superoxide anion and the activity of the superoxide dismutase enzyme.

The positive adsorption of melatonin onto the microspheres resulted in a uniformly spherical morphology, with an increase in size when colostrum melatonin was adsorbed, which was confirmed through flow cytometry. Various scientific studies have widely recognized the development of systems that enable the modified release of compounds from new polymers and the use of established polymers in innovative formulations [17,18]. Microspheres made from polymeric substances can be utilized for the controlled delivery of drugs, plant-active components, and hormones [6,18,19].

This study’s fluorescence microscopy and flow cytometry analysis revealed that the adsorption of melatonin extracted from colostrum in PEG microspheres increased the size of microspheres. In contrast, synthetic melatonin reduced the size of the microspheres. Melatonin is a small hydrophobic molecule, and its interaction with the PEG matrix can vary based on several factors, including the degree of crosslinking, the hydrophobicity of the polymer, and the distribution of hydrophilic and hydrophobic domains within the microsphere. When melatonin adsorbs onto PEG microspheres, it can disrupt the polymer matrix, leading to an increase in size due to enhanced hydrophilicity or changes in the packing of the polymer. Conversely, if melatonin interacts strongly with the PEG matrix, it may promote crosslinking or tighten the polymer structure, decreasing its diameter [20,21]. These findings align with previous studies that observe variable properties of PEG microspheres depending on the type of bioactive substance [6,22] and can explain the changes in the diameter of the microspheres when adsorbed with colostrum-derived or synthetic melatonin.

Polyethylene glycol (PEG) in microsphere formulations can facilitate the control of pore development by adjusting molecular weight and concentration. It can also regulate the rate at which drugs or hormones are released from the polymer matrix [4,6,7,18]. In our study, we found that melatonin adsorbed onto PEG microspheres exhibited a satisfactory release profile in terms of both concentration and time. Previous research has shown similar results, confirming synthetic melatonin release from a polymer matrix [6].

The effectiveness of hormones combined with a polymeric matrix has expanded the potential for developing new drugs and enhancing cellular activation in the presence of bacteria or tumor cells [6,23]. Melatonin, in particular, is a hormone that directly stimulates the immune system and boosts cellular metabolism. The production of free radicals is recognized as an important protective mechanism during infectious processes, the possibility of obtaining new drugs, and the capacity for cellular activation [6,22]. Melatonin is a hormone that directly stimulates the immune system and cellular metabolism by generating free radicals, which has been considered an important protection mechanism during infectious processes [13].

The results of this study confirm the role of superoxide anion in the activation mechanisms of colostrum phagocytes, particularly in modified drug release systems. Additionally, the findings emphasize the differences between melatonin extracted from colostrum and synthetic melatonin, especially regarding their interaction with enteropathogenic *Escherichia coli* (EPEC).

Both types of melatonin, extracted from colostrum or synthetic, increased the release of superoxide anion by phagocytes in the absence of bacteria. However, when synthetic melatonin was absorbed into PEG microspheres, the superoxide release decreased, while colostrum-extracted melatonin continued to enhance this release. These findings suggest that synthetic melatonin encapsulated in PEG functions as an antioxidant, neutralizing reactive oxygen species and protecting cells from oxidative stress. In contrast, melatonin derived from colostrum may stimulate cellular processes that temporarily boost the production of reactive oxygen species. Controlled release of superoxide can reduce adverse effects, which is particularly important in infectious processes.

During oxidative metabolism, peroxisomal and mitochondrial processes release excessive amounts of superoxide anion. Consequently, in infections, the body produces free radicals, which are key strategies for protection [23,24]. However, the body has an efficient antioxidant defense system with various enzymes [25,26]. Superoxide dismutase (SOD) is an enzyme that plays a crucial role in the human body’s antioxidant defense system [2,10,27]. In this study, melatonin extracted from colostrum adsorbed onto PEG microspheres in the presence of bacteria, resulting in a reduction in the release of the superoxide dismutase (SOD) enzyme and a decrease in the SOD/O_2_^−^ ratio. In contrast, synthetic melatonin increased both the release of the superoxide dismutase enzyme and the SOD/O_2_^−^ ratio, suggesting that both hormones alter the balance between the pro- and antioxidant systems differently.

Reactive oxygen species (ROS) can function as signaling molecules that regulate various cellular processes, including the self-destruction of bacteria [28,29,30]. However, when there is an imbalance between the levels of ROS and the cell’s ability to eliminate them, oxidative stress occurs [31,32]. This study observed that treating colostrum phagocytes with synthetic melatonin, whether encapsulated in PEG microspheres or not, increased the oxidative index. This treatment led to a rise in superoxide dismutase (SOD) levels and a decrease in superoxide anion, reinforcing the antioxidant properties of melatonin in its synthetic form.

Colostrum melatonin, regardless of its adsorption to PEG microspheres, has been shown to stimulate the release of superoxide, which is significant for infectious processes. However, it also leads to a reduction in the oxidative index, indicating an improved balance between superoxide anion production and the stimulation of superoxide dismutase (SOD) in a more efficient manner. Additionally, colostrum contains various bioactive and immunomodulatory compounds that can enhance antioxidant and pro-oxidant effects [33,34,35] and tend to act without triggering inflammatory processes [34]. Therefore, colostrum melatonin appears more effective in balancing cellular oxidative metabolism, likely due to its natural origin and interaction with other components in the secretion.

The study highlights the importance of melatonin’s source and formulation in oxidative cellular metabolism and underscores the roles of immunology and antioxidant therapies in infection treatment. Colostrum-derived melatonin enhances superoxide production while reducing SOD activity, creating a pro-oxidant environment that helps fight infections. Encapsulating melatonin in PEG microspheres enables targeted delivery to phagocytes, enhancing immune responses without harming healthy tissues. It also emphasizes how the biological characteristics of compounds in modified-release systems can influence their immunomodulatory effects. The structural and functional characterization of PEG microspheres underlines the significance of using polymers as controlled release systems, thereby contributing to the advancement of pharmaceutical technologies to optimize treatments.

Melatonin administered via polyethylene glycol (PEG) microspheres holds promise for various clinical applications [6,36]. Newborns, especially premature infants, have underdeveloped immune systems, making them more susceptible to infections and inflammation. Adjuvant therapy can enhance immune responses in severe infections without high antibiotic doses, reducing the risk of resistance [37,38]. Melatonin from colostrum, when incorporated into infant formulas, can mimic the protective benefits of breast milk, offering immunity support for those not breastfed. Additionally, the hormone can serve in antioxidant therapies for patients facing inflammatory diseases [17,39,40]. This technology could help clinical practice by enabling a safe and targeted approach to neonatal care, immune modulation, and inflammatory conditions. Controlling melatonin release using PEG microspheres could transform the treatment of inflammatory diseases and infections by providing a more targeted and safe approach, particularly for vulnerable populations like neonates and individuals with chronic conditions, potentially preventing damage from chronic inflammation and minimizing adverse effects on healthy tissues.

The effectiveness of hormones can vary depending on factors such as the specific system used, the components present in colostrum, and the quantity of melatonin extracted. Due to its neuroprotective and antioxidant properties, melatonin combined with PEG microspheres shows potential for treating chronic diseases related to oxidative stress, such as autoimmune diseases, respiratory and cardiovascular diseases, neurodegenerative disorders, and cancer. However, research is needed to confirm the effectiveness of colostrum-derived melatonin in various preclinical and clinical models, particularly regarding its interactions with phagocytes and their effects on immunological and metabolic parameters. Clinical studies are essential to ensure safety and efficacy, and personalizing treatments for controlled release will be important for managing oxidative metabolism and immune responses effectively.

## 5. Conclusions

The results of this study suggest that melatonin can be extracted from colostrum. This hormone was found to adsorb onto polyethylene glycol (PEG) microspheres. The adsorption of melatonin increased the size of the microspheres and demonstrated the potential to modulate the functional activity of phagocytes present in colostrum.

Functional evaluations demonstrated that melatonin, whether extracted or synthetic, can stimulate the release of superoxide by phagocytes and influence the concentration of the enzyme superoxide dismutase (SOD). Additionally, it suggests that melatonin derived from colostrum is more effective than synthetic. This finding supports the developed system’s immunomodulatory role in the interaction between melatonin and phagocytes from colostrum and the hormone’s potential for therapeutic use in treating bacterial infections.

## Figures and Tables

**Table 1:** Analysis of polyethylene glycol microspheres by flow cytometry.

Microsphere	Size (µm)	Fluorescence Intensity (Mean ± sd)
BD	6	95.1 ± 9.3
PEG	5.1	80.3 ± 10.7
PEG + MLT-C	6.8	107.2 ± 12.9
PEG + MLT-S	5.4	84.1 ± 13.6

Notes: BD—Becton Dickinson—polymethylmethacrylate microspheres; PEG—Polyethylene glycol microspheres; Polyethylene glycol microspheres with colostrum-derived melatonin (MLT-C); and Polyethylene glycol microspheres with synthetic melatonin (MLT-S).

## Data Availability

The authors will make the data supporting this study’s interpretations available if requested.
